# Adult Attachment Affects Neural Response to Preference-Inferring in Ambiguous Scenarios: Evidence From an fMRI Study

**DOI:** 10.3389/fpsyg.2018.00139

**Published:** 2018-03-06

**Authors:** Xing Zhang, Guangming Ran, Wenjian Xu, Yuanxiao Ma, Xu Chen

**Affiliations:** ^1^Faculty of Psychology, Southwest University, Chongqing, China; ^2^Key Laboratory of Cognition and Personality, Southwest University, Chongqing, China; ^3^Institute of Education, China West Normal University, Nanchong, China

**Keywords:** preference-inferring, theory of mind, adult attachment, fMRI, anterior insula, inferior parietal lobule

## Abstract

Humans are highly social animals, and the ability to cater to the preferences of other individuals is encouraged by society. Preference-inferring is an important aspect of the theory of mind (TOM). Many previous studies have shown that attachment style is closely related to TOM ability. However, little is known about the effects of adult attachment style on preferences inferring under different levels of certainty. Here, we investigated how adult attachment style affects neural activity underlying preferences inferred under different levels of certainty by using functional magnetic resonance imaging (fMRI). The fMRI results demonstrated that adult attachment influenced the activation of anterior insula (AI) and inferior parietal lobule (IPL) in response to ambiguous preference-inferring. More specifically, in the ambiguous preference condition, the avoidant attached groups exhibited a significantly enhanced activation than secure and anxious attached groups in left IPL; the anxious attached groups exhibited a significantly reduced activation secure attached group in left IPL. In addition, the anxious attached groups exhibited a significantly reduced activation than secure and avoidant attached groups in left AI. These results were also further confirmed by the subsequent PPI analysis. The results from current study suggest that, under ambiguous situations, the avoidant attached individuals show lower sensitivity to the preference of other individuals and need to invest more cognitive resources for preference-reasoning; while compared with avoidant attached group, the anxious attached individuals express high tolerance for uncertainty and a higher ToM proficiency. Results from the current study imply that differences in preference-inferring under ambiguous conditions associated with different levels of individual attachment may explain the differences in interpersonal interaction.

## Introduction

“Different strokes for different folks” implies that every individual has a preference for a specific object or activity. Human beings are highly social groups and the ability to cater to the preferences of other individuals is encouraged in society. Consequently, the ability to accurately infer the preferences of another individual is critical for a successful social interaction in daily life. The ability of preference-inferring based on intuition involves theory of mind (ToM) (Gore and Sadler-Smith, 2011), and the ToM refers to the ability to infer other individuals’ mental states and to make predictions about their behavior. Preference-inferring is an important aspect of the ToM. In daily life, we may encounter situations associated with different levels of certainty. In some situations, it is possible to infer the preferences of other individuals easily by analyzing the available information. For example, we may come to the conclusion that Xiaoming likes to eat fish by adopting the rule that consistent selection of an object over other alternatives indicates preference for that object (Kelley, 1987). However, many inferences regarding the human mind (e.g., preference) are ambiguous. For example, if Liming buys orange juice on some occasions and coke on others, it is impossible to infer preference for a particular drink. As the available information in ambiguous situations is too limited to make inferences, the observers must proceed with a provisional hypothesis about the mental states of the target, which remain ambiguous until further information is obtained. Gilbert (1998) indicated that observers did make assumptions about the mental states of other individuals under ambiguous circumstances, but they did not do so using the rule-based approach that is used in case of conditions of greater certainty. Simulationist theory, one of the theories that could explain how an individual endeavors to understand the mind of another, suggests that, although observers have not direct access to the mind of another individual, they can directly access the conscious experiences of their own mind, thus, they could use their own conscious experience as an available model to comprehend the mental of that individual (Gordon, 1986). Given a scarcity of available information, preference-inferring under ambiguous scenarios may rely more on self-simulated and internally formed information, which may arise from their own firsthand experiences or similar circumstances in the past (Jenkins and Mitchell, 2009). Therefore, the self-based simulationist approach could be used to understand the mind of others under ambiguous scenarios.

Many previous studies (Hari et al., 1998; Iacoboni et al., 1999; Oberman et al., 2005) have indicated that the mirror-neuron system (MNS) of human is mainly analogously involved two cortical areas, namely, inferior frontal cortex (IFG) and inferior parietal lobule (IPL). MNS is found to be closely related with imitative behavior (Iacoboni, 2005) and social cognition (Iacoboni et al., 2005). The MNS, especially IPL, is closely associated with comprehension of intention and imitation. For example, robust activity was found in IPL in monkeys when they engaged in a task which needed them to infer the experimenter’ intentions (Fogassi et al., 2005). Neuroimaging evidence from human studies also reported significant activity in IPL during imitation tasks condition (Chaminade et al., 2002; Nakamura et al., 2004) and ToM task (Kobayashi et al., 2007). Given that the MNS is strongly associated with comprehension of intention and imitation, and inferring preferences under ambiguous conditions relies more on simulated, internally generated information, it is reasonable to deduce that there may be significant activity in MNS while subjects are engaged in inferring the mental state of other individuals (e.g., preference) under ambiguous conditions. In addition, the insular cortex, particularly anterior insula (AI), was found to be involved in processing empathy and uncertainty (Singer et al., 2009). In addition, evidence from findings in non-human primates also showed that the AI and especially in its anterior-basal parts have been found to be involved in the emotion processing and empathy processing (Frith and Singer, 2008; Lamm and Singer, 2010). As preference carry considerable emotional component, preference-inferring can be viewed as affective ToM. Some researchers suggested that the concept of ‘affective ToM’ was very similar to that of empathy (Shamay-Tsoory et al., 2005). Furthermore, the schizophrenia patients tend to have deficits in social cognition, and the results from different ToM paradigms found reduced activation in insula among schizophrenia patients (Russell et al., 2000; Brüne et al., 2008), implying that insula cortex was engaged in inferring the mental state of other individuals. More importantly, studies on decision-making have reported increased signal in the AI in response to ambiguity (Huettel et al., 2006). Since the preferences of other individuals, in ambiguous scenarios, are associated with high degree of uncertainty, and given that the insula cortex involves in ToM and empathy, it is reasonable to deduce that there may be significant activity in AI while participants are engaged in inferring preferences under ambiguous condition.

Attachment theory offers an important framework for interpersonal interaction experiences that play a critical role in regulating affect, cognition, and interpersonal behavior, and are also related with interpersonal functioning, resilience, and mental symptoms (Bentall et al., 2007; Berry et al., 2008; Rutten et al., 2013). According to the attachment theory, interactions with early main caregivers are memorized and organized as schematic, or script-like internal working models (Bowlby, 1982), which then lay the foundation of the nature of an individual’s attachment style. The responsive and trustful experiences of interaction with caregivers (i.e., attachment figure) lead to the formation of a secure attachment style. However, insensitive, unsupportive, or dismissive experiences with attachment figures lead individuals to develop an insecure attachment style (Hazan and Shaver, 1987). Insecure attachment mainly includes avoidant attachment and anxious attachment. The avoidant attachment is marked by “deactivation strategies,” such as maintaining self-reliance, and tending to avoid emotional states that could activate their attachment system. However, the anxious attachment is marked by “hyperactivation strategies,” such as heightened attention to threat-related thoughts and memories (Mikulincer and Shaver, 2007).

The attachment style of an individual remains relatively stable into adulthood, and provides a schema for determining how people perceive and react during a variety of social encounters. As an important schema of interpersonal interaction, attachment style affects ToM abilities. ToM skills are considered to more likely to develop within secure attachment bonds (Fonagy and Target, 1997), implying that, attachment style may have an influence on ToM, which also has been supported by many other studies (Humfress et al., 2002). In addition, a recent study, which investigated the relationship between ToM and attachment anxiety and avoidance among adolescence, found that anxiety toward the mother was associated with inaccurate mindreading (Hünefeldt et al., 2013b). This indicates that insecure individuals may be deficient in ToM abilities. However, adopting the same ToM paradigm, Hünefeldt et al. (2013a) found that anxious attached women may be better mindreader when stimuli were emotionally neutral or difficult to recognize. It is still unknown whether adult insecure attachment has a negative impact on ToM abilities. More importantly, to the best of our known, there is only one neuroimaging study has investigated the effect of neural basis underlying adult attachment style on ToM processes (Schneider-Hassloff et al., 2015). Furthermore, although preference-inferring is an important aspect of ToM, there is no study has explored the effect of adult attachment on preference-inferring. Furthermore, since the preferences of other individuals have an emotional component, understanding, and catering to these preferences contributes to the formation intimate relationships. Compared with secure attached adults, the insecure attached adults tend to exhibit greater difficulty in establishing or maintaining intimate relationships (McCarthy and Maughan, 2010). Thus, the inability to accurately understand and cater to the preferences of other individuals may be related to the poor quality or lack of intimate relationships of insecure individuals during adulthood. Therefore, investigating the neural mechanisms behind the effect of adult attachment on preference-inferring under unambiguous and ambiguous conditions may contribute us better understanding about the relationship between the relationship quality and adult attachment.

We were aiming to explore the potential neural mechanisms underlying the effect of adult attachment on preference-inferring under unambiguous and ambiguous conditions. Participants were scanned using functional magnetic resonance imaging (fMRI) while they read short vignettes that described an individual’s preferences under unambiguous or ambiguous condition. Based on the results of previous studies mentioned above, we anticipate that adult attachment style influences neural activity of MNS-associated brain regions and AI when individuals infer preferences of others, especially in ambiguous conditions.

## Materials and Methods

### Participants

A total of 436 Chinese undergraduate students were selected for online administration of the Chinese version of the Experience in Close Relationships Scale (ECR), and the Relationship Questionnaire (RQ) (Tonggui and Kazuo, 2006). According to screening criteria of previous studies (Chavis and Kisley, 2012; Ma et al., 2017a,b), Twenty participants (4.6%), scoring lower than 1 standard deviation (*SD*) below the mean on both attachment-avoidance and attachment-anxiety dimensions, and classified as a secure individual by the RQ, were assigned to the “secure attachment” group (nine males; mean age = 21.15 years, *SD* = 1.50). Twenty participants (4.6%) with ECR scores higher than 1 *SD* above the attachment avoidance dimension and lower than that for the attachment anxiety dimension, and classified as an dismissing individual by the RQ, were assigned to the “avoidant attachment” group (eight males; mean age = 19.85 years, *SD* = 1.53). Eighteen participants (4.1%) with ECR scores higher than 1 *SD* above the attachment anxiety dimension and lower than that for the attachment avoidance dimension, and classified as an preoccupied individual by the RQ, were assigned to the “anxious attachment” group (11 males; mean age = 20.28 years, *SD* = 1.93). All participants were right-handed, reported normal or corrected-to-normal vision, and had no history of neurological disorders. Participants signed written informed consent forms and were financially compensated for their participation. The data were analyzed anonymously, and personal identification information was handled confidentially. The study was approved by the local ethics committee and the methods were carried out in accordance with the Helsinki guidelines as per the WHO (Gilder, 1964).

### Measurements

A Chinese version of the ECR and the RQ were used in the current study. The validity and reliability of these tools have been consistently demonstrated in the Chinese population. The scale consists of two dimensions: the attachment anxiety (e.g., “I worry about being abandoned”) and attachment avoidance (e.g., “I prefer not to show a partner how I feel deep down”). The two dimensions are composed of 18 items each. Participants were required to rate the extent to which each item described their experiences in close relationships, on a seven-point rating scale ranging from 1 (not at all) to 7 (very much). The scale achieved reliable internal consistency for both attachment anxiety (0.928) as well as attachment avoidance (0.930).

The RQ describes four attachment prototypes (secure attachment, preoccupied attachment, dismissing attachment, and fearful attachment), using four paragraphs, based on self/other representation dimensions (Bartholomew and Horowitz, 1991). Participants were instructed to rate the extent to which each prototype described their experience on a seven-point scale, and the highest prototype score determined the individual’s attachment style. Li and Kato (2006) have earlier reported a strong negative correlation between the attachment avoidance score of the ECR and the other-representation score of the RQ, as well as the attachment anxiety score of the ECR and the self-representation score of the RQ. According to previous studies (Griffin and Bartholomew, 1994; Debbané et al., 2017), the self-model scores was calculated by the rating scores of prototype featured by a positive view of the self (i.e., secure and dismissing) minus the rating scores of patterns featured by a negative view of self (i.e., the fearful and preoccupied). The other model scores was computed by the rating scores for prototype characterized by a positive view of others (i.e., secure and preoccupied) minus rating scores for patterns characterized by a negative view of others (i.e., fearful and dismissing). In current study, there was significant negative correlation between the attachment avoidance score of the ECR and the other-representation score of the RQ (*r* = -0.49, *p* < 0.01), as well as the attachment anxiety score of the ECR and the self-representation score of the RQ (*r* = -0.48, *p* < 0.01).

### Preference-Inferring Task

The short vignettes used in current study were translated from those used in previous study (Jenkins and Mitchell, 2009). The short vignettes contain three kinds of scenarios, namely, unambiguous preferences scenarios, ambiguous preferences scenarios, and non-social scenarios. We invited English major post-graduates to translate the English vignettes to Chinese, and adjusted the content on the basis of differences between Chinese and western cultures. In order to ensure the effectiveness of the translated vignettes, we conducted one pilot experiment before the actual fMRI experiment. Thirty-three subjects (16 males; mean age = 21.32, *SD* = 1.54) read the three kinds of vignette and answered the questions. Subjects were also required to rate the answers in terms of unambiguity, using a five-point scale: 1 (very ambiguous) to 5 (very unambiguous). The data obtained indicated that the unambiguous preference scenarios and non-social scenarios were understood well, the percentages of correct understanding were 88.88 and 75.24%, respectively. The unambiguous preference scenarios and ambiguous preference scenarios differed significantly in their unambiguity of answer [unambiguous preferences scenarios: 4.3 ± 0.34, ambiguous preferences scenarios: 2.51 ± 0.64; *F*(1,64) = 14.30; *p* < 0.0001], and the non-social scenarios and ambiguous preferences scenarios also differed significantly in their unambiguity of answer [unambiguous preferences scenarios: 4.07 ± 0.44, ambiguous preferences scenarios: 2.51 ± 0.64; *F*(1,64) = 11.51; *p* < 0.0001]. Therefore, the short vignettes could be well-understood by Chinese subjects, and were fit to be used as experimental materials.

During scanning, participants read short vignettes relating everyday events. The vignettes mainly described a protagonist’s preferences. In the unambiguous version of each scenario, the preferences of the protagonist would be strongly suggested, but not explicitly stated. In the ambiguous versions of each scenario, the protagonist’s preferences could be any one of multiple possibilities under the circumstances, that is, the information provided left the inference more open-ended. Following each preference scenario, participants needed to answer a single multiple-choice question about the protagonist’s preferences; while following each non-social scenario, participants needed to answer a question about a physical representation (**Table 1**). After each trial started, the story and the question were presented together on screen for a total of 10 s. This was followed by four response choices, which were presented for 4 s. During this interval, participants were instructed to select their chosen ending by a key-press response. This was followed by 12 s of fixation [inter-stimulus interval (ISI)]. Each participant completed a total of 24 preference scenarios and 12 non-social scenarios across 2 run, with the presentation randomized across participants, and no participants encountering both ambiguous and unambiguous versions of the same story. During the course of the experiment, subjects lay in the supine position in the MRI scanner, holding a box with buttons used to indicate the response. The accuracy and reaction time (RT) were recorded while subjects performed the task, using the same software that was used for presenting stimulus (Presentation, Neurobehavioral Systems, Albany, CA, United States).

**Table 1:** Example experimental item in each of the three conditions.

Conditions	Scenarios	Questions
Unambiguous preference	Xiaoming went to the movies three times during the summer vacation.	Where does Xiaoming prefer to sit in a theater?
	He and his friends arrived early and sat in the front of the theater every time.	(1) In the front
		(2) In the middle
		(3) In the back
		(4) Somewhere else
Ambiguous preference	Xiaoming went to the movies three times during the summer vacation.	Where does Carl prefer to sit in a theater?
	He and his friends arrived late and sat in the front of the theater every time.	(1) In the front
		(2) In the middle
		(3) In the back
		(4) Somewhere else
Non-social	This part of the garden is supposed to be reserved for the roses it’s labeled accordingly. Recently, the garden was deserted, and dandelions have taken over the entire flower bed.	What does the label say the flowers are?
		(1) Dandelions
		(2) Roses
		(3) Chinese rose
		(4) Something else

### fMRI Data Acquisition

Brain images were collected using a 3T Siemens scanner (Siemens Magnetom Trio TIM, Erlangen, Germany). Functional images were acquired using a gradient echo-planar imaging (EPI) sequence (repetition time [TR] = 2000 ms, echo time [TE] = 30 ms, flip angle [FA] = 90°, field of view [FoV] = 192 mm × 192 mm, matrix size = 64 × 64 pixels, voxel size = 3 mm × 3 mm × 3 mm, inter-slice skip = 0.99 mm, and number of slices = 32). T1-weighted images consisted of 176 slices that were 1 mm thick, with an in-plane resolution of 0.98 mm × 0.98 mm (TR = 1900 ms; TE = 2.52 ms; FA = 9°; FOV = 250 mm × 250 mm; voxel dimensions = 1 mm × 1 mm × 1 mm).

### fMRI Data Analysis

Functional image processing was preprocessed using Statistical Parametric Mapping (SPM8^1^) and Data Preprocessing Assistant for Resting-state FMRI (DPARSF^2^). They were slice-time- corrected, realigned to the first image, and corrected for head movements. The anatomical images were then co-registered to the mean EPI images and segmented into white matter, gray matter, and cerebrospinal fluid (CSF). The EPI images were then spatially normalized to the Montreal Neurological Institute (MNI) space with the structural information from co-registration and segmentation. Subsequently, the acquired images were spatially smoothed using one 8 mm full-width-at-half-maximum (FWHM) Gaussian kernel.

The general linear model (GLM) was implemented in SPM8 to identify blood-oxygen-level dependency (BOLD) activation in relation to separate event types. On the first level, an event-related design was used, with three types of events: unambiguous preference, ambiguous preference, non-social control. Each event was convolved (time locked to the onset of each scenario) with a canonical hemodynamic response function (HRF), and a high-pass temporal filter (cut off at 128 s) was applied. Each trial was modeled as a separate event (duration = 14). Six regressors, representing movement-related variance, were also employed in the design matrix. The first level analysis of each participant yielded three individual contrast images (unambiguous preference > non-social control, unambiguous preference; ambiguous preference > non-social control, ambiguous preference; unambiguous preference > ambiguous preference, unambiguous effect) that described the parameter estimates associated with each event modeled. The resulting contrast images were then entered into separate second-level analyses for contrasts of interest, where attachment style (secure attachment, avoidant attachment, anxious attachment) served as a between-subjects variable in a full-factorial ANOVA. For the interaction analysis, the average percentage of change in signal was extracted from the significant clusters for each condition using MarsBar (Brett et al., 2002) to examine the direction of the response, and the SPSS 16.0 was used to conduct a simple effect analysis. Correction for multiple comparisons was performed using Monte Carlo simulation. The statistical threshold used for these data was set to *p* < 0.05 (one-tailed, uncorrected) at the individual voxel level. According to previous researches in ToM domains (Wang et al., 2015) and attachment domains (Tang et al., 2017), we used AlphaSim program in DPABI software (Yan et al., 2016) to correct for multiple comparisons. According to the assumption that meaningful activation in fMRI was spatially clustered, Forman et al. (1995) suggested that the approach of combining voxel probability threshold with a non-arbitrary minimum cluster size threshold can protect against false positives (Type 1 error). Therefore, statistical significance was set at a combined threshold of *p* < 0.001 and a minimum cluster size of 46 voxels, which corresponded to a corrected threshold of *p* < 0.05 (AlphaSim corrected) using the updated version in the DPABI V2.3 toolkit^3^. The bug in AlphaSim correction reported by Eklund et al. (2016) has been overcome in the DPABI. The following parameters were used to perform the Alphasim correction: single voxel *p* = 0.001, 10000 simulations, voxel size = 3 mm × 3 mm × 3 mm, voxels in mask = 51775, estimated smoothness of statistical map = 11.076 mm × 11.602 mm × 11.047 mm.

### Functional Connectivity Analysis

In the present study, we use a supplementary psychophysiological interaction (PPI) analysis to explore whether the changes in connectivity between a seed region of interest (AI and IPL) and other regions depend on specific experimental condition (psychological factors). Based on the BOLD activation results (the seed regions were defined by the survived brain regions in the attachment group × preference condition interaction), we defined the AI and IPL as seed regions with the contrasts of interest (unambiguous preference minus non-social control: unambiguous preference; ambiguous preference minus non-social control: ambiguous preference; unambiguous preference minus ambiguous preference: unambiguous effect), and extracted the participant-specific time course of activity in the left AI and IPL with a 4-mm radius sphere centered at the coordinates of the two-way interaction. Separate first-level analyses for “unambiguous preference,” “ambiguous preference,” and “unambiguous effect” were performed for each seed. In order to explore the effect of attachment patterns on task-dependent connectivity of the seeds, first-level contrast of the PPI regressors for “unambiguous preference,” “ambiguous preference,” and “unambiguous effect” were fed into separate whole brain 3 × 3 full-factorial ANOVA models for each brain region (i.e., left AI and IPL). Therefore, we created two 3 × 3 full-factorial ANOVA models comprising the factor “attachment style” (three levels: “secure attachment,” “anxious attachment,” and “avoidant attachment”) and the factor “preference condition” (three levels: “unambiguous preference,” “ambiguous preference,” and “unambiguous effect”) resulting in nine cells. In the second-level analysis of PPI, clusters were determined using a significant threshold of *p* < 0.001 uncorrected at a voxel-wise whole-brain level, and a cluster size of *k* > 40 contiguous voxels are presented.

## Results

### Behavioral Data Analysis

For each group, mean reaction times for correct trials and percentage accuracy rates were averaged across the participants. These data are displayed in **Table 2**. An analysis of the preference condition (unambiguous preference, non-social control) by group (secure attachment, anxious attachment, avoidant attachment) repeated-measures ANOVA, with mean RT as the dependent variable, revealed a significant main effect of preference condition [*F*(1,55) = 103.98, *p* < 0.0001], indicating that participants responded faster to unambiguous preference (1984.38 ± 210.42 ms) than non-social control (2950.13 ± 326.92 ms). While the interaction between preference condition and attachment group was not significant [*F*(2,55) = 0.261, *p* = 0.77 (>0.05)]. A preference condition (unambiguous preference, non-social control) by group (secure attachment, anxious attachment, avoidant attachment) repeated-measures ANOVA, with percentage accuracy as the dependent variable, revealed a significant main effect of preference condition[*F*(1,55) = 83.20, *p* < 0.0001], indicating that participants responded more accurately in unambiguous preference condition (88.07 ± 11.90) than in non-social control (68.25 ± 16.11), while the interaction between preference condition and attachment group was not significant [*F*(2,55) = 0.05, *p* = 0.95 (>0.05)].

**Table 2:** Means and standard deviations for percentage accuracy and reaction time (RT) data for the preference condition (unambiguous preference and non-social control), presented by Condition and Group.

	Secure	Anxious	Avoidant
**Mean RT (SD)**			
Unambiguous preference	1581.71 (169.57)	1556.71 (269.67)	1508.19 (189.92)
Non-social control	1949.20 (336.49)	1932.52 (399.35)	1827.35 (235.83)
**Percentage accuracy (SD)**			
Unambiguous preference	87.92 (13.38)	87.50 (12.86)	88.75 (9.86)
Non-social control	68.33 (15.20)	66.67 (18.08)	69.58 (15.83)

*RT, response time; SD, standard deviation.*

### Analysis of Neuroimaging Results

#### Main Effect of Preference

The analysis revealed that the left IFG (MNI -45 24 30), left middle frontal gyrus (MNI -45 6 54), and superior frontal gyrus (MNI 0 18 54) survived by contrasting ambiguous preference reasoning with unambiguous preference reasoning (**Table 3**). No suprathreshold activation was associated with the opposite contrast.

**Table 3:** Main effect of preference.

Regions	BA	MNI			*z*-value	Voxels
		*x*	*y*	*z*		
Unambiguous > Ambiguous						
No activated clusters						
Ambiguous > Unambiguous						
L Inferior/middle frontal gyrus	9	-45	24	30	3.75	221
L Middle frontal gyrus	6	-45	6	54	3.94	52
L Superior/medial frontal gyrus	6	0	18	54	3.51	53

*Coordinates (mm) are in MNI space. L, left hemisphere.*

#### Main Effect of Attachment Style

The analysis revealed that left IPL (MNI -60 -33 36), left middle frontal gyrus (MNI -45 42 21) survived by contrasting avoidant attachment group with secure attachment group; right middle temporal gyrus (MNI 69 -9 -9) and left superior frontal gyrus (MNI -9 54 30) survived by contrasting secure attachment group with avoidant attachment group. In addition, the analysis revealed that no suprathreshold was associated with the contrast between anxious attachment group and secure attachment group (**Table 4**), while activations in left IFG (MNI -54 21 6), left superior temporal gyrus (MNI -57 -63 18), left middle temporal gyrus (MNI -51 -9 -27), left middle temporal gyrus (MNI 69 -21 -18) and left superior temporal gyrus (MNI -51 -18 -3) was associated with the opposite contrast. No suprathreshold activation was associated with the contrast between anxious attachment group and avoidant group, as well as the opposite contrast.

**Table 4:** Main effect of attachment style.

Regions	BA	MNI			*z*-value	Voxels
		*x*	*y*	*z*		
Avoidant > Secure						
L Inferior parietal lobule	40	-60	-33	36	4.47	63
L Middle frontal gyrus	46	-45	42	21	3.81	20
Secure > Avoidant						
R Middle temporal gyrus	21	69	-9	-9	4.40	57
L Superior frontal gyrus	9	-9	54	30	4.20	56
Secure > Anxious						
L Inferior frontal gyrus	45	-54	21	6	4.28	48
L Inferior frontal gyrus	47	-39	30	-9	4.08	20
L Superior/middle temporal gyrus	39	-57	-63	18	4.03	96
L Middle/superior temporal gyrus	21	-51	-9	-27	4.02	103
L Middle temporal gyrus	21	69	-21	-18	3.77	63
L Middle/superior temporal gyrus	21	-51	-18	-3	3.76	37
Anxious > Secure						
No activated clusters						
Anxious > Avoidant						
No activated clusters						
Avoidant > Anxious						
No activated clusters						

*Coordinates (mm) are in MNI space. L, left hemisphere; R, right hemisphere.*

#### Interaction Between Attachment Group and Preference Condition

**FIGURE 1:**
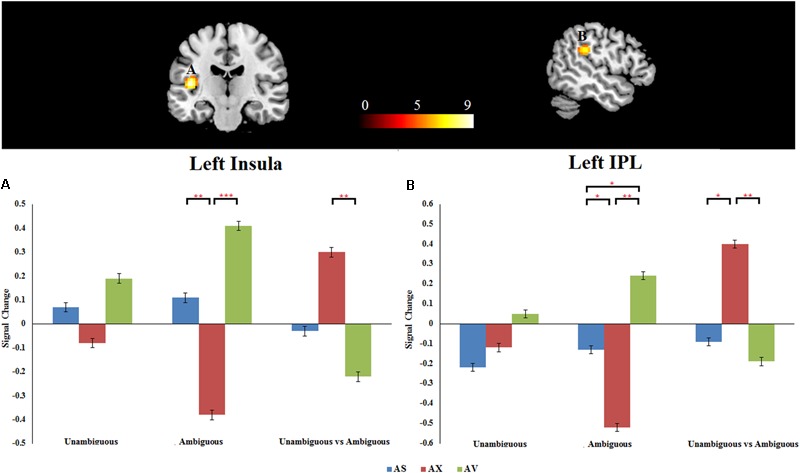
**(A)** Left insula. **(B)** Left IPL. Attachment group × Preference condition. Displayed is a rendering of (Attachment group × Preference condition) interaction from the factorial analysis. Bar chart displays the percent signal change in secure attachment group (blue), anxious attachment group (red) and avoidant attachment group (green) for unambiguous, ambiguous and unambiguous vs. ambiguous conditions. Detailed results can be found in **Table 5**. The asterisks (^∗^) indicate significant differences (^∗^*p* ≤ 0.05, ^∗∗^*p* ≤ 0.01, ^∗∗∗^*p* ≤ 0.001).

As mentioned above, the primary interest of the current study was to examine whether the attachment pattern would affect preference-inferring. The attachment group × preference condition interaction revealed two significant activation clusters (AlphaSim corrected, *p* < 0.001, **Table 5**) in the left IPL (MNI -51 -33 27) and left AI (MNI -42 -21 12). The average percentage of change in signal was extracted from left IPL and AI to determine the nature of this interaction (**Figure 1**). For the contrast (ambiguous preference vs. non-social control), the anxious attached groups exhibited a significantly reduced activation than secure attached groups in left AI (*p* = 0.007); the anxious attached groups exhibited a significantly reduced activation than avoidant attached groups in left AI (*p* = 0.000); the avoidant attached groups exhibited a significantly enhanced activation than secure attached groups in left IPL (*p* = 0.022); the avoidant attached groups exhibited a significantly enhanced activation than anxious attached groups in left IPL (*p* = 0.000); the anxious attached groups showed a significantly reduced activation than secure attached groups in left IPL (*p* = 0.022). For the contrast (unambiguous preference vs. ambiguous preference), a simple effect analysis indicated that anxious attached groups exhibited a significantly enhanced activation than avoidant attached groups in left AI (*p* = 0.003); the anxious attached groups exhibited a significantly enhanced activation than secure attached groups in left IPL (*p* = 0.014); the anxious attached groups exhibited a significantly enhanced activation than avoidant attached groups in left IPL (*p* = 0.004).

**Table 5:** Regions for interaction between attachment group and preference condition at *p* < 0.001 (cluster > 90, Alphasim 0.001 corrected).

Regions	BA	MNI			*z*-value	Voxels
		*x*	*y*	*z*		
Interaction: attachment group × ToM condition						
L Insula/superior temporal gyrus	13	-42	-21	12	4.79	122
L Inferior parietal lobule	40	-51	-33	27	4.15	123

*Coordinates (mm) are in MNI space. L, left hemisphere.*

#### Effects of Functional Connectivity

**FIGURE 2:**
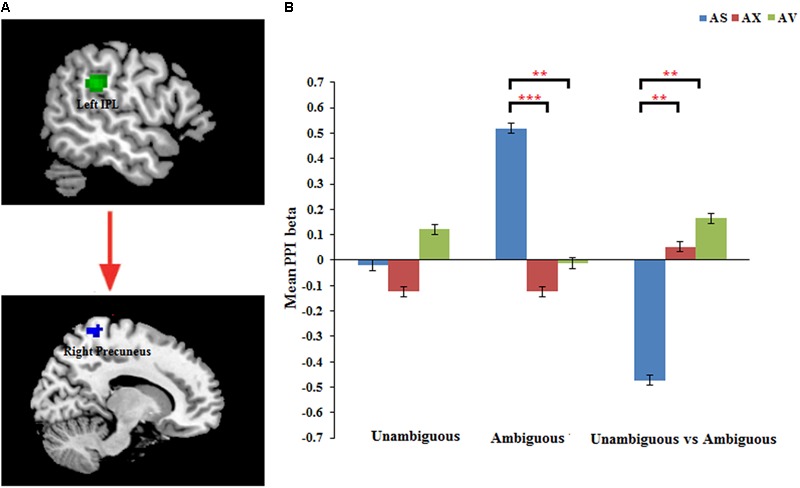
**(A)** The interaction between attachment group and Preference condition for left IPL in right precuneus. **(B)** Bar chart displays the mean PPI beta in secure attachment group (blue), anxious attachment group (red) and avoidant attachment group (green) for unambiguous, ambiguous and unambiguous vs. ambiguous conditions. Detailed results can be found in **Table 6**. The asterisks (^∗^) indicate significant differences (^∗∗^*p* ≤ 0.01, ^∗∗∗^*p* ≤ 0.001).

**FIGURE 3:**
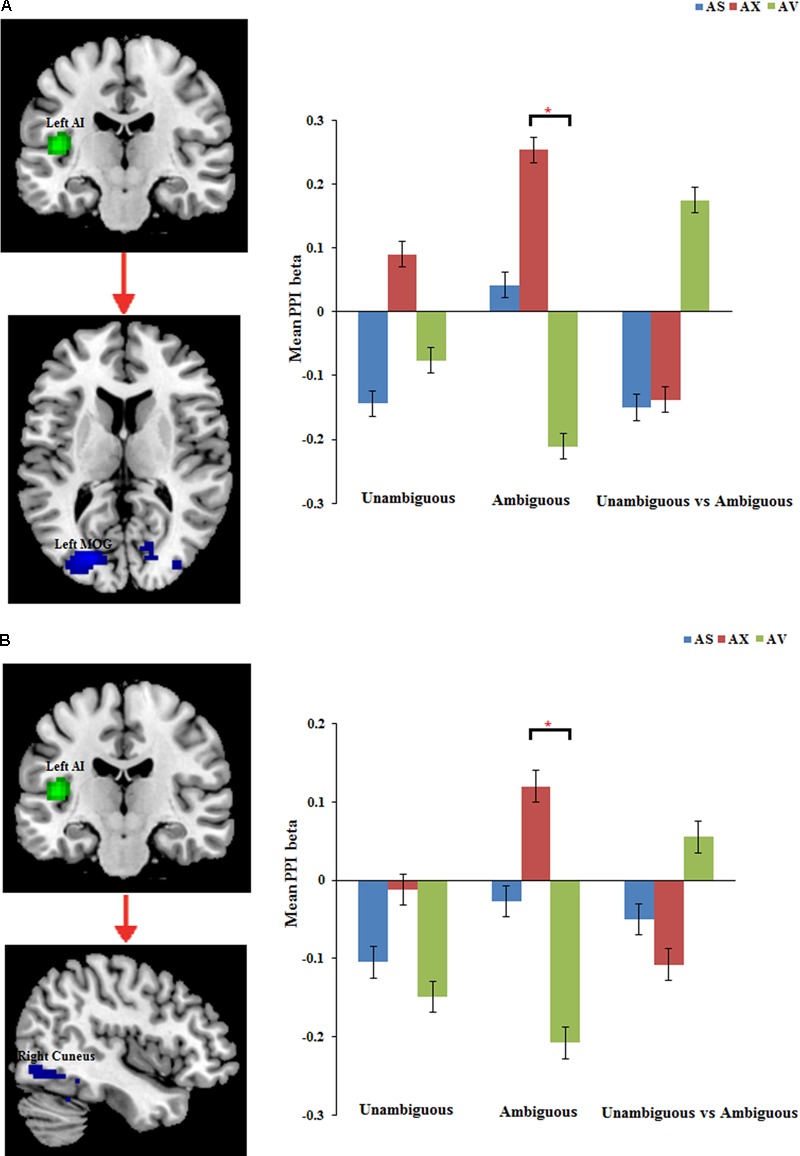
The regions forinteraction between attachment group and Preference condition for left AI. Bar chart displays the mean PPI beta in secure attachment group (blue), anxious attachment group (red) and avoidant attachment group (green) for unambiguous, ambiguous and unambiguous vs. ambiguous conditions. **(A)** Left AI connectivity with MOG/Fusiform gyrus. **(B)** Left AI connectivity with Cuneus/Posterior cingulate. Detailed results can be found in **Table 6**. The asterisks (^∗^) indicate significant differences (^∗^*p* ≤ 0.05).

The PPI analysis showed that a significant interaction effect of preference condition by group on left IPL connectivity with right precuneus (*z* = 4.16, *p* < 0.001, uncorrected, see **Table 6**). After conducting further analysis, we found that, compared with insecure attached group (anxious and avoidant attached group), the secure attached group showed a stronger functional connectivity between the left IPL with the right precuneus during ambiguous preference condition (**Figure 2**). While for the contrast (unambiguous preference vs. ambiguous preference), the secure attached group showed weaker functional connectivity between the left IPL and the right precuneus than insecure attached groups. In addition, the PPI analysis revealed a significant interaction effect of preference condition by group on left AI connectivity with left middle occipital gyrus (MOG) extending to fusiform gyrus and inferior occipital gyrus (IOG) (*z* = 4.75, *p* < 0.001, uncorrected), right cuneus extending to posterior cingulate gyrus (PCG) and middle temporal gyrus (MTG) (*z* = 3.80, *p* < 0.001, uncorrected). After conducting further analysis, we found that, compared with avoidant group, the anxious group showed a stronger functional connectivity between the left AI and the left MOG during ambiguous preference condition (**Figure 3A**); compared with avoidant group, the anxious group also showed a stronger functional connectivity between the left AI and the right cuneus during ambiguous preference condition (**Figure 3B**).

**Table 6:** Results of the interaction between attachment group and preference condition for left IPL and AI connectivity.

Regions	BA	MNI			*z*-value	Voxels
		*x*	*y*	*z*		
Interaction effects of IPL connectivity						
R Precuneus	7	15	-48	72	4.16	45
Interaction effects of AI connectivity						
L Middle occipital gyrus/fusiform gyrus/inferior occipital gyrus/cuneus	18	-21	-81	12	4.75	937
R Cuneus/middle occipital gyrus/posterior cingulate/middle temporal gyrus	30	30	-72	21	3.80	110

*Coordinates (mm) are in MNI space. L, left hemisphere; R, right hemisphere.*

## Discussion

The current study employed a text-based ToM task to explore how adult attachment orientations influence neural activity when participants infer the preferences of other individuals, under unambiguous and ambiguous conditions. Interestingly, we found that adult attachment orientations influence neural activity in response to ambiguous preference-inferring in left IPL and AI (extending to superior temporal gyrus). Specifically, in the ambiguous preference condition, the avoidant attached groups exhibited a significantly enhanced activation than secure attached groups in left IPL; the anxious attached groups exhibited a significantly reduced activation than avoidant and secure attached group in left IPL. We also found that the anxious attached groups exhibited a significantly reduced activation than secure attached groups in left AI; the anxious attached groups also exhibited a significantly reduced activation than avoidant attached groups in left AI. In addition, we compared the activation of unambiguous and ambiguous preference-inferring, which was named “unambiguous effect.” We found that the “unambiguous effect” of anxious attached group was significantly greater than that of avoidant attached groups in left AI, and was also significantly greater than secure attached groups and avoidant attached groups in left IPL. The results from PPI analysis revealed that the secure attached group showed a stronger functional connectivity between the left IPL with the right precuneus than insecure attached groups during ambiguous preference condition; compared with avoidant group, the anxious group showed a stronger functional connectivity between the left AI and the left MOG, right cuneus during ambiguous preference condition. The findings from PPI analysis provide further evidence for activating findings.

Our text-based ToM neuroimaging study demonstrated left lateralized brain activity, which is in line with previous studies (Gallagher et al., 2000). Estimation of the preferences of other individuals might be influenced by personality traits (Kang et al., 2013). The results obtained in this study confirm this, and further indicate that adult attachment affects the process of preference-inferring in ambiguous situations. It has been mentioned that the IPL is associated with intention inference (Fogassi et al., 2005) and imitation (Nakamura et al., 2004). In addition, the IPL has also been found to be associated with executive function (Barch and Csernansky, 2007; Torrey, 2007), enhanced activation of IPL may reflect more cognitive resources involved in cognitive task. The present fMRI data indicate that the avoidant attached groups exhibited a significantly enhanced activation than secure attached groups and anxious attached groups in left IPL, while engaged in ambiguous preference-inferring task. The findings are consistent with the hypothesis. The ability to judge personal traits of other individuals could reflect individuals’ interpersonal sensitivity (Ambady et al., 2001). Since avoidant attachment is associated with constant effort to maintain feelings of autonomy and self-reliance (Mikulincer and Shaver, 2007), the avoidant attachment individuals tend to have a negative perception of other individuals, and consequently are less sensitive to signs of rejection or acceptance. Therefore, the enhanced activation of IPL in avoidant attachment group may reflect the characteristics of low sensitivity when inferring others’ preferences in ambiguous situations, and they need to invest more cognitive resources for preference-inferring. The finding that attachment traits affect neural responses in IPL during ambiguous preference-inferring also supports previous results indicating decision-making under uncertainty is supported by IPL (Huettel et al., 2005; Vickery and Jiang, 2009).

It is very interesting to found that, in addition to left IPL, the anxious attached groups exhibited a significantly reduced activation than secure and avoidant attached groups in left AI (extending to superior temporal gyrus) during the ambiguous preference condition. Evidences from affective neuroscience and social neuroscience have demonstrated that insular cortex, particularly AI, plays an especially role in empathy processing and uncertainty processing (Singer et al., 2009). The degree to which participants could understand their own emotions was found to be associated with activity in the AI during an interoceptive task (Silani et al., 2008). Increased activity of AI has been linked to intolerance of uncertainty while processing affectively ambiguous faces (Simmons et al., 2008), and the insula cortex also involved in the maintenance of anxiety (Paulus and Stein, 2006). Thus, the responsivity of AI may reflect the tolerance of uncertainty and the level of anxiety. In addition, Brüne et al. (2008) found that the insula was more activated in healthy subjects than patients with schizophrenia under ToM condition. This study suggests that the insula may act a role in the processing of ToM. Given that AI or insula also play an important role in many cognitive processes (e.g., interoception, pain processing and attention), the role that AI plays in the processing of ToM may not be prominent, while the extending brain area (i.e., superior temporal gyrus) is the ToM-related region and plays a critical role in the processing of ToM. The superior temporal gyrus has been found to have a particular role in understanding causality and intentionality (Frith and Frith, 2001), thus involving in the processing of ToM (Völlm et al., 2006). Furthermore, a study which examined the relationship between adult attachment and ToM, found that women’s attachment anxiety was related with better mind reading when the stimuli were difficult to recognize (Hünefeldt et al., 2013a), implying that the anxious attached individuals may have higher affective mentalizing ability in relatively difficult ToM task. Preference-inferring under ambiguous condition is also a relatively difficult ToM task. Preference carry considerable emotional component, our inferences about preference of other individuals may be guided by the nature of our own experience, especially under ambiguous situation. Given that anxious attached individual performed better in relatively difficult ToM task. A low responsivity of the AI to preference-inferring under ambiguous conditions may imply anxious attached groups’ high tolerance for uncertainty and low anxiety, which may further reflect a higher ToM proficiency among anxious attached individuals under ambiguous condition.

In addition, the fMRI data demonstrated that the “unambiguous effect” of anxious attached group was significantly greater than avoidant attached groups in left AI, and was significantly greater than secure attached groups and avoidant attached groups in left IPL. The anxious attached individuals appear to activate their attachment behavioral system more easily which is closely related with a tendency of hypervigilance toward social-emotional stimuli (Bartholomew and Horowitz, 1991). The value associated with self for anxiously attached individuals is especially dependent on the approval and evaluation of other individuals (Park et al., 2004). Understanding the preferences of other individuals and catering to them may be particularly important for anxious individuals. As mentioned above, the IPL and insula were involved in processing empathy and uncertainty (Huettel et al., 2005; Singer et al., 2009; Vickery and Jiang, 2009). The “unambiguous effect” of anxious attached group was significantly greater than other attached groups in left AI and IPL, and may imply that the processing modes of unambiguous preference and ambiguous preference among anxious attached group were different from other attached groups. In order to clarify the mechanism of the differences, further research may needed in the future.

Our findings of the differential effects of adult attachment orientations on neural activity in response to ambiguous preference-inferring in left IPL and AI are further supplemented by PPI analysis showing different patterns of functional connectivity to left IPL and AI during ambiguous preference-inferring. Specifically, during ambiguous preference-inferring, the functional connectivity between left IPL and right precuneus was stronger in the secure attached group than that in the other two insecure groups. The precuneus has been related to episodic memory retrieval (Addis et al., 2004; Cavanna and Trimble, 2006) and self-processing (Cavanna and Trimble, 2006). The episodic memory entails the recollection of information that is linked to an individual’s personal experiences. In order to understand others’ mental state, successful ToM reasoning needs individuals to imagine the mindset of others and simulate their experience (Blakemore and Decety, 2001), which may also involve recalling personal past experiences, especially in ambiguous situations. During the ambiguous preference-inferring, in order to infer the protagonist’s preference more effectively, the subjects may need to recall their personal experiences in similar situations. Furthermore, compared with insecure attached individuals, the memory extraction of secure attached individuals is more smooth (Dykas et al., 2010). Therefore, the finding that the functional connectivity between left IPL and right precuneus was stronger in the secure attached group than that in the other two insecure groups during ambiguous preference-inferring, may imply that, when inferring others’ preferences in ambiguous situation, the secure individuals are more likely to recall the personal experiences, which may in turn lead them to perform well. In addition, the PPI analysis from current study showed that the functional connectivity between left AI and MOG (extending to fusiform gyrus and inferior occipital gyrus) was stronger in the anxious attached group than that in the avoidant attached group during ambiguous preference-inferring. Moreover, there was also stronger functional connectivity between left AI and cuneus (extending to posterior cingulate and middle temporal gyrus) in the anxious attached group than that in the avoidant attached group during ambiguous preference-inferring. The occipital areas (e.g., fusiform gyrus) was found to be involved in the processing of the visual properties of Chinese characters and words (Tan et al., 2000), word recognition (Proverbio et al., 2008) and preference procesing (Vartanian and Goel, 2004). While the cuneus/posterior cingulate has been found to be involved in self-referential processing (Fossati et al., 2003), and plays a pivotal role in the default model network (Fransson and Marrelec, 2008). The default model network was found to be associated with ToM reasoning (Spreng and Grady, 2010). Furthermore, evidences from resting state connectivity found an emotional salience monitoring system which linking the AI with cingulate gyrus (Taylor et al., 2009). Although the anxious attached group showed a low responsivity of the AI to preference-inferring under ambiguous preference-inferring, they showed much stronger functional connectivity between left AI and MOG, cuneus/posterior cingulate, the brain regions involved in preference processing or self-referential processing, and the stronger functional connectivity may contribute to better performance during ambiguous preference-inferring.

### Limitations

Although current study has obtained some interesting findings, several limitations need to be taken into account. First of all, it may be due to the fact that the experimental trials are relatively few or there may be other moderators that play a role in the association between attachment style and preferences inferring, we did not found significant interaction between attachment style and preferences inferring in terms of either RT or accuracy. In order to clarify the mechanisms behind the effects of adult attachment style on preferences inferring, more study need to be conducted in the future. Secondly, although the Alphasim correction has been widely used in fMRI study, compared with other stricter multiple correction methods (e.g., family-wise error), Alphasim correction is a relatively loose multiple comparison correction method, we may need make cautious inference for the results.

## Conclusion

The present study was conducted to investigate the effect of adult attachment orientations among healthy young adults on the neural response to preference-inferring under unambiguous and ambiguous conditions. Adult attachment orientations were found to moderate the activation of AI and IPL in response to ambiguous preference-inferring, which was also further confirmed by the subsequent PPI analysis. Our results also indicated that the “unambiguous effect” of anxious attached group was significantly greater than other attached groups in left AI and IPL. Our findings indicate that the avoidant attached individuals show lower sensitivity to the preference of other individuals and need to invest more cognitive resources for preference-reasoning, especially in ambiguous situations. Compared with avoidant attached group, the anxious attached individuals express high tolerance for uncertainty and a higher ToM proficiency during ambiguous preference-reasoning. The findings from current study help to our better understanding of the neural basis behind the effect of adult attachment on the process of preference-inferring. Understanding and catering to the preferences of other individuals is crucial for interpersonal interaction. Our findings imply that differences in preference-inferring under ambiguous conditions among different attachment individuals may explain the differences in performance during interpersonal interactions.

## Ethics Statement

All participants provided written informed consent prior to the study. The study was approved by the Institutional Human Participants Review Board of Southwest University Imaging Center for Brain Research.

## Author Contributions

XC and XZ designed the experiments. XZ and GR carried out the experiments. XZ, WX, and YM analyzed the experimental results. XZ wrote the manuscript.

## Conflict of Interest Statement

The authors declare that the research was conducted in the absence of any commercial or financial relationships that could be construed as a potential conflict of interest.

## Footnotes

1 http://www.fil.ion.ucl.ac.uk/spm

2 http://rfmri.org/DPARSF

3 http://rfmri.org/dpabi

## References

[B1] Addis D. R. McIntosh A. R. Moscovitch M. Crawley A. P. McAndrews M. P. (2004). Characterizing spatial and temporal features of autobiographical memory retrieval networks: a partial least squares approach. *Neuroimage* 23 1460–1471. 10.1016/j.neuroimage.2004.08.007 15589110

[B2] Ambady N. LaPlante D. Johnson E. (2001). “Thin-slice judgments as a measure of interpersonal sensitivity,” in *Interpersonal Sensitivity: Theory and Measurement* eds Hall J. A. Bernieri F. J. (Mahwah, NJ: Erlbaum) 89–101.

[B3] Barch D. M. Csernansky J. G. (2007). Abnormal parietal cortex activation during working memory in schizophrenia: verbal phonological coding disturbances versus domain-general executive dysfunction. *Am. J. Psychiatry* 164 1090–1098. 10.1176/ajp.2007.164.7.1090 17606661

[B4] Bartholomew K. Horowitz L. M. (1991). Attachment styles among young adults: a test of a four-category model. *J. Pers. Soc. Psychol.* 61 226–244. 10.1037/0022-3514.61.2.226 1920064

[B5] Bentall R. P. Fernyhough C. Morrison A. P. Lewis S. Corcoran R. (2007). Prospects for a cognitive-developmental account of psychotic experiences. *Br. J. Clin. Psychol.* 46 155–173. 10.1348/014466506X123011 17524210

[B6] Berry K. Barrowclough C. Wearden A. (2008). Attachment theory: a framework for understanding symptoms and interpersonal relationships in psychosis. *Behav. Res. Ther.* 46 1275–1282. 10.1016/j.brat.2008.08.009 18926521

[B7] Blakemore S. J. Decety J. (2001). From the perception of action to the understanding of intention. *Nat. Rev. Neurosci.* 2 561–567. 10.1038/35086023 11483999

[B8] Bowlby J. (1982). Attachment and loss: retrospect and prospect. *Am. J. Orthopsychiatry* 52 664–678. 10.1111/j.1939-0025.1982.tb01456.x 7148988

[B9] Brett M. Anton J. L. Valabregue R. Poline J. B. (2002). Region of interest analysis using the MarsBar toolbox for SPM 99. *Neuroimage* 16:S497.

[B10] Brüne M. Lissek S. Fuchs N. Witthaus H. Peters S. Nicolas V. (2008). An fMRI study of theory of mind in schizophrenic patients with ”passivity” symptoms. *Neuropsychologia* 46 1992–2001. 10.1016/j.neuropsychologia.2008.01.023 18329671

[B11] Cavanna A. E. Trimble M. R. (2006). The precuneus: a review of its functional anatomy and behavioural correlates. *Brain* 129 564–583. 10.1093/brain/awl004 16399806

[B12] Chaminade T. Meltzoff A. N. Decety J. (2002). Does the end justify the means? A PET exploration of the mechanisms involved in human imitation. *Neuroimage* 15 318–328. 10.1006/nimg.2001.0981 11798268

[B13] Chavis J. M. Kisley M. A. (2012). Adult attachment and motivated attention to social images: attachment-based differences in event-related brain potentials to emotional images. *J. Res. Pers.* 46 55–62. 10.1016/j.jrp.2011.12.004 22639475 PMC3359649

[B14] Debbané M. Badoud D. Sander D. Eliez S. Luyten P. Vrtièka P. (2017). Brain activity underlying negative self- and other-perception in adolescents: the role of attachment-derived self-representations. *Cogn. Affect. Behav. Neurosci.* 17 554–576. 10.3758/s13415-017-0497-9 28168598 PMC5403860

[B15] Dykas M. J. Woodhouse S. S. Ehrlich K. B. Cassidy J. (2010). Do adolescents and parents reconstruct memories about their conflict as a function of adolescent attachment? *Child Dev.* 81 1445–1459. 10.1111/j.1467-8624.2010.01484.x 20840233 PMC2941233

[B16] Eklund A. Nichols T. E. Knutsson H. (2016). Cluster failure: why fMRI inferences for spatial extent have inflated false-positive rates. *Proc. Natl. Acad. Sci. U.S.A.* 113 7900–7905. 10.1073/pnas.1602413113 27357684 PMC4948312

[B17] Fogassi L. Ferrari P. F. Gesierich B. Rozzi S. Chersi F. Rizzolatti G. (2005). Parietal lobe: from action organization to intention understanding. *Science* 308 662–667. 10.1126/science.1106138 15860620

[B18] Fonagy P. Target M. (1997). Attachment and reflective function: their role in self-organization. *Dev. Psychopathol.* 9 679–700. 10.1017/S0954579497001399 9449001

[B19] Forman S. D. Cohen J. D. Fitzgerald M. Eddy W. F. Mintun M. A. Noll D. C. (1995). Improved assessment of significant activation in functional magnetic resonance imaging (fMRI): use of a cluster-size threshold. *Magn. Reson. Med.* 33 636–647. 10.1002/mrm.1910330508 7596267

[B20] Fossati P. Hevenor S. J. Graham S. J. Grady C. Keightley M. L. Craik F. (2003). In search of the emotional self: an fMRI study using positive and negative emotional words. *Am. J. Psychiatry* 160 1938–1945. 10.1176/appi.ajp.160.11.1938 14594739

[B21] Fransson P. Marrelec G. (2008). The precuneus/posterior cingulate cortex plays a pivotal role in the default mode network: evidence from a partial correlation network analysis. *Neuroimage* 42 1178–1184. 10.1016/j.neuroimage.2008.05.059 18598773

[B22] Frith C. D. Singer T. (2008). The role of social cognition in decision making. *Philos. Trans. R. Soc. Lond.* 363 3875–3886. 10.1098/rstb.2008.0156 18829429 PMC2581783

[B23] Frith U. Frith C. (2001). The biological basis of social interaction. *Curr. Dir. Psychol. Sci.* 10 151–155. 10.1111/1467-8721.00137

[B24] Gallagher H. L. Happé F. Brunswick N. Fletcher P. C. Frith U. Frith C. D. (2000). Reading the mind in cartoons and stories: an fMRI study of ‘theory of mind’ in verbal and nonverbal tasks. *Neuropsychologia* 38 11–21. 10.1016/S0028-3932(99)00053-6 10617288

[B25] Gilbert D. T. (1998). “Ordinary personology,” in *Handbook of Social Psychology* eds Gilbert D. T. Fiske S. T. Lindzey G. (New York: McGraw Hill) 89–150.

[B26] Gilder S. (1964). World medical association meets in Helsinki. *Br. Med. J.* 2 299–300. 10.1136/bmj.2.5404.299 20790244 PMC1815581

[B27] Gordon R. M. (1986). Folk psychology as simulation. *Mind Lang.* 1 158–171. 10.1111/j.1468-0017.1986.tb00324.x

[B28] Gore J. Sadler-Smith E. (2011). Unpacking intuition: a process and outcome framework. *Rev. Gen. Psychol.* 15 304–316. 10.1037/a0025069

[B29] Griffin D. Bartholomew K. (1994). Models of the self and other: fundamental dimensions underlying measures of adult attachment. *J. Pers. Soc. Psychol.* 67 430–445. 10.1037/0022-3514.67.3.430 15896922

[B30] Hari R. Forss N. Avikainen S. Kirveskari E. Salenius S. Rizzolatti G. (1998). Activation of human primary motor cortex during action observation: a neuromagnetic study. *Proc. Natl. Acad. Sci. U.S.A.* 95 15061–15065. 10.1073/pnas.95.25.15061 9844015 PMC24575

[B31] Hazan C. Shaver P. (1987). Romantic love conceptualized as an attachment process. *J. Pers. Soc. Psychol.* 52 511–524. 10.1037/0022-3514.52.3.511 3572722

[B32] Huettel S. A. Song A. W. Mccarthy G. (2005). Decisions under uncertainty: probabilistic context influences activation of prefrontal and parietal cortices. *J. Neurosci.* 25 3304–3311. 10.1523/JNEUROSCI.5070-04.2005 15800185 PMC6724903

[B33] Huettel S. A. Stowe C. J. Gordon E. M. Warner B. T. Platt M. L. (2006). Neural signatures of economic preferences for risk and ambiguity. *Neuron* 49 765–775. 10.1016/j.neuron.2006.01.024 16504951

[B34] Humfress H. O’connor T. G. Slaughter J. Target M. Fonagy P. (2002). General and relationship-specific models of social cognition: explaining the overlap and discrepancies. *J. Child Psychol. Psychiatry* 43 873–883. 10.1111/1469-7610.0013_7 12405476

[B35] Hünefeldt T. Laghi F. Ortu F. (2013a). Are anxiously attached women better mindreaders? *Cogn. Process.* 14 317–321. 10.1007/s10339-013-0556-2 23529700

[B36] Hünefeldt T. Laghi F. Ortu F. Belardinelli M. O. (2013b). The relationship between ‘theory of mind’ and attachment-related anxiety and avoidance in Italian adolescents. *J. Adolesc.* 36 613–621. 10.1016/j.adolescence.2013.03.012 23595130

[B37] Iacoboni M. (2005). Neural mechanisms of imitation. *Curr. Opin. Neurobiol.* 15 632–637. 10.1016/j.conb.2005.10.010 16271461

[B38] Iacoboni M. Molnar-Szakacs I. Gallese V. Buccino G. Mazziotta J. C. Rizzolatti G. (2005). Grasping the intentions of others with one’s own mirror neuron system. *PLoS Biol.* 3:e79. 10.1371/journal.pbio.0030079 15736981 PMC1044835

[B39] Iacoboni M. Woods R. P. Brass M. Bekkering H. Mazziotta J. C. Rizzolatti G. (1999). Cortical mechanisms of human imitation. *Science* 286 2526–2528. 10.1126/science.286.5449.2526 10617472

[B40] Jenkins A. C. Mitchell J. P. (2009). Mentalizing under uncertainty: dissociated neural responses to ambiguous and unambiguous mental state inferences. *Cereb. Cortex* 20 404–410. 10.1093/cercor/bhp109 19478034 PMC2803737

[B41] Kang P. Lee J. Sul S. Kim H. (2013). Dorsomedial prefrontal cortex activity predicts the accuracy in estimating others’ preferences. *Front. Hum. Neurosci.* 7:686. 10.3389/fnhum.2013.00686 24324419 PMC3840299

[B42] Kelley H. H. (1987). Attribution in social interaction. *Paper Presented at the Preparation of this Grew Out of a Workshop on Attribution Theory Held at University of California, Los Angeles, August 1969* (Lawrence: Erlbaum Associates, Inc.).

[B43] Kobayashi C. Glover G. H. Temple E. (2007). Children’s and adults’ neural bases of verbal and nonverbal ‘theory of mind’. *Neuropsychologia* 45 1522–1532. 10.1016/j.neuropsychologia.2006.11.017 17208260 PMC1868677

[B44] Lamm C. Singer T. (2010). The role of anterior insular cortex in social emotions. *Brain Struct. Funct.* 214 579–591. 10.1007/s00429-010-0251-3 20428887

[B45] Li T. Kato K. (2006). Measuring adult attachment: Chinese adaptation of the ECR scale. *Acta Psychol. Sin.* 38 399–406.

[B46] Ma Y. Chen X. Ran G. Ma H. Zhang X. Liu G. (2017a). The processing of body expressions during emotional scenes: the modulation role of attachment styles. *Sci. Rep.* 7:44740. 10.1038/srep44740 28303949 PMC5356188

[B47] Ma Y. Ma H. Chen X. Ran G. Zhang X. (2017b). Do attachment patterns predict aggression in a context of social rejection? An executive functioning account. *Aggress. Behav.* 43 408–418. 10.1002/ab.21700 28168707

[B48] McCarthy G. Maughan B. (2010). Negative childhood experiences and adult love relationships: the role of internal working models of attachment. *Attach. Hum. Dev.* 12 445–461. 10.1080/14616734.2010.501968 20730639

[B49] Mikulincer M. Shaver P. R. (2007). *Attachment in Adulthood: Structure, Dynamics, and Change*. New York City, NY: Guilford Press.

[B50] Nakamura A. Maess B. Knösche T. R. Gunter T. C. Bach P. Friederici A. D. (2004). Cooperation of different neuronal systems during hand sign recognition. *Neuroimage* 23 25–34. 10.1016/j.neuroimage.2004.04.034 15325349

[B51] Oberman L. M. Hubbard E. M. McCleery J. P. Altschuler E. L. Ramachandran V. S. Pineda J. A. (2005). EEG evidence for mirror neuron dysfunction in autism spectrum disorders. *Cogn. Brain Res.* 24 190–198. 10.1016/j.cogbrainres.2005.01.014 15993757

[B52] Park L. E. Crocker J. Mickelson K. D. (2004). Attachment styles and contingencies of self-worth. *Pers. Soc. Psychol. Bull.* 30 1243–1254. 10.1177/0146167204264000 15466598

[B53] Paulus M. P. Stein M. B. (2006). An insular view of anxiety. *Biol. Psychiatry* 60 383–387. 10.1016/j.biopsych.2006.03.042 16780813

[B54] Proverbio A. M. Zani A. Adorni R. (2008). The left fusiform area is affected by written frequency of words. *Neuropsychologia* 46 2292–2299. 10.1016/j.neuropsychologia.2008.03.024 18485421

[B55] Russell T. A. Rubia K. Bullmore E. T. Soni W. Suckling J. Brammer M. J. (2000). Exploring the social brain in schizophrenia: left prefrontal under activation during mental state attribution. *Am. J. Psychiatry* 157 2040–2042. 10.1176/appi.ajp.157.12.2040 11097974

[B56] Rutten B. P. Hammels C. Geschwind N. Mennelothmann C. Pishva E. Schruers K. (2013). Resilience in mental health: linking psychological and neurobiological perspectives. *Acta Psychiatr. Scand.* 128 3–20. 10.1111/acps.12095 23488807 PMC3746114

[B57] Schneider-Hassloff H. Straube B. Nuscheler B. Wemken G. Kircher T. (2015). Adult attachment style modulates neural responses in a mentalizing task. *Neuroscience* 303 462–473. 10.1016/j.neuroscience.2015.06.062 26162239

[B58] Shamay-Tsoory S. G. Tomer R. Berger B. D. Goldsher D. Aharon-Peretz J. (2005). Impaired “affective theory of mind” is associated with right ventromedial prefrontal damage. *Cogn. Behav. Neurol.* 18 55–67. 10.1097/01.wnn.0000152228.90129.99 15761277

[B59] Silani G. Bird G. Brindley R. Singer T. Frith C. Frith U. (2008). Levels of emotional awareness and autism: an fMRI study. *Soc. Neurosci.* 3 97–112. 10.1080/17470910701577020 18633852

[B60] Simmons A. Matthews S. C. Paulus M. P. Stein M. B. (2008). Intolerance of uncertainty correlates with insula activation during affective ambiguity. *Neurosci. Lett.* 430 92–97. 10.1016/j.neulet.2007.10.030 18079060 PMC2692473

[B61] Singer T. Critchley H. D. Preuschoff K. (2009). A common role of insula in feelings, empathy and uncertainty. *Trends Cogn. Sci.* 13 334–340. 10.1016/j.tics.2009.05.001 19643659

[B62] Spreng R. N. Grady C. L. (2010). Patterns of brain activity supporting autobiographical memory, prospection, and theory of mind, and their relationship to the default mode network. *J. Cogn. Neurosci.* 22 1112–1123. 10.1162/jocn.2009.21282 19580387

[B63] Tan L. H. Spinks J. A. Gao J. H. Liu H. L. Perfetti C. A. Xiong J. (2000). Brain activation in the processing of Chinese characters and words: a functional MRI study. *Hum. Brain Mapp.* 10 16–27. 10.1002/(SICI)1097-0193(200005)10:1<16::AID-HBM30>3.0.CO;2-M 10843515 PMC6871809

[B64] Tang Q. Chen X. Hu J. Liu Y. (2017). Priming the secure attachment schema affects the emotional face processing bias in attachment anxiety: an fMRI research. *Front. Psychol.* 8:624. 10.3389/fpsyg.2017.00624 28473796 PMC5398122

[B65] Taylor K. S. Seminowicz D. A. Davis K. D. (2009). Two systems of resting state connectivity between the insula and cingulate cortex. *Hum. Brain Mapp.* 30 2731–2745. 10.1002/hbm.20705 19072897 PMC6871122

[B66] Tonggui L. Kazuo K. (2006). Measuring adult attachment: Chinese adaptation of the ECR scale. *Acta Psychol. Sin.* 38 399–406.

[B67] Torrey E. F. (2007). Schizophrenia and the inferior parietal lobule. *Schizophr. Res.* 97 215–225. 10.1016/j.schres.2007.08.023 17851044

[B68] Vartanian O. Goel V. (2004). Neuroanatomical correlates of aesthetic preference for paintings. *Neuroreport* 15 893–897. 10.1097/00001756-200404090-00032 15073538

[B69] Vickery T. J. Jiang Y. V. (2009). Inferior parietal lobule supports decision making under uncertainty in humans. *Cereb. Cortex* 19 916–925. 10.1093/cercor/bhn140 18728197

[B70] Völlm B. A. Taylor A. N. Richardson P. Corcoran R. Stirling J. McKie S. (2006). Neuronal correlates of theory of mind and empathy: a functional magnetic resonance imaging study in a nonverbal task. *Neuroimage* 29 90–98. 10.1016/j.neuroimage.2005.07.022 16122944

[B71] Wang Y. Liu W. Li Z. Wei X. Jiang X. Neumann D. L. (2015). Dimensional schizotypy and social cognition: an fMRI imaging study. *Front. Behav. Neurosci.* 9:133. 10.3389/fnbeh.2015.00133 26074796 PMC4444828

[B72] Yan C. G. Wang X. D. Zuo X. N. Zang Y. F. (2016). DPABI: data processing & analysis for (resting-state) brain imaging. *Neuroinformatics* 14 339–351. 10.1007/s12021-016-9299-4 27075850

